# Loss of Bace1 in Mice Does Not Alter the Severity of Caerulein Induced Pancreatitis

**DOI:** 10.1371/journal.pone.0125556

**Published:** 2015-05-11

**Authors:** Mario Heindl, Jan Tuennemann, Ines Sommerer, Joachim Mössner, Albrecht Hoffmeister

**Affiliations:** Department of Internal Medicine, University Hospital of Leipzig, Leipzig, Germany; Klinikum rechts der Isar der TU München, GERMANY

## Abstract

**Context:**

Beta-site alpha-amyloid protein cleaving enzyme1 (BACE1) plays a key role in the pathogenesis of Alzheimer’s disease. Additional to its moderate expression in the brain, high levels of BACE1 mRNA were found in the pancreas. Murine Bace1 has been immunohistochemicaly detected at the apical pole of acinar cells within the exocrine pancreas of mice and Bace1 activity was observed in pancreatic juice. In vitro experiments revealed enteropeptidase as a putative substrate for Bace1 suggesting a role in acute pancreatitis.

**Objective:**

The aim of this study was to address a protective mechanism of Bace1 in acute experimental pancreatitis in mice.

**Methods:**

Acute experimental pancreatitis was induced by intraperitoneal injection of caerulein in homozygote *Bace1* ^-/-^ mice and wild type mice. Serum and tissue analyses were carried out after 4 h, 8 h and 24 h. Measurement of plasma amylase and lipase was performed to confirm pancreatitis induction. In order to assess the severity of pancreatitis H&E stained pancreatic sections were examined regarding edema, inflammation and apoptosis. Immunohistochemical detection of myeloperoxidase (MPO) positive cells was carried out to further quantify the extent of inflammation. Expression of Bace2 within the pancreas was analyzed by immunohistochemistry and RT-qPCR.

**Results:**

We demonstrate that total loss of Bace1 in mice leads to no alterations in the course of acute experimental caerulein-pancreatitis. *Bace1^-/-^* mice develop a moderate pancreatitis that is comparable in histomorphological and serological features with those seen in wild type mice.

**Discussion:**

We discuss the results in the context of the applied caerulein induced edematous pancreatitis model and possible compensatory mechanisms via Bace2 that might be responsible for the observed results.

## Introduction

Acute pancreatitis is considered to be an abdominal emergency. Although most episodes are mild and self-limiting, the overall mortality of acute pancreatitis is still 5–10%. Despite the advances in intensive care, mortality can increase up to 30%, if complications like extensive necrosis develop [1]. The fatal course of the disease is mostly owing to systemic inflammatory response syndrome and multi-organ failure [2]. Up to now, the pathogenic mechanisms of acute pancreatitis are not well understood.

The pancreas secretes most of the digestive enzymes, such as trypsinogen as inactive zymogens [3]. The concept of acinar cell injury due to premature trypsinogen activation has been supported by several data, both from experimental pancreatitis and human hereditary pancreatitis [4–8]. Physiologically, trypsin is secreted as its inactive precursor zymogen, trypsinogen, which will be activated by cleavage through enteropeptidase in the intestinal lumen [3]. Although controversially discussed, active trypsin reflux due to high pancreatic duct pressure, uncontrolled trypsin activation and exocrine pancreatic hyperstimulation are thought to play an important role in triggering autodigestion of acinar cells. Acute pancreatitis may develop, in case of failure of intracellular protective mechanisms which prevent premature trypsinogen activation or reduce trypsin activity [9].

The aspartic endoprotease beta-site amyloid precursor protein-cleaving enzyme1 (BACE1) was first identified in 1999 as a key player in the generation of beta-amyloid peptides, the major constituent of brain plaques in patients with Alzheimer’s disease [10–13]. Although BACE1 is characterized as a type I transmembrane aspartic protease [13], it has been detected in the cerebrospinal fluid [14], suggesting an additional function besides intracellular amyloidogenesis in the brain. BACE1 mRNA was found at rather high levels in pancreatic tissue [15, 16]. We recently reported that Bace1 is expressed in the exocrine pancreas of mice and located especially at the apical pole of acinar cells. Interestingly, Bace1 activity was not only found in the pancreas but also in the pancreatic juice. Furthermore, enteropeptidase—the main activator of trypsin in the intestinal lumen—was indentified as one putative substrate for BACE1 *in vitro* [17]. So far, the physiological function of BACE1 and its substrates within the exocrine pancreas are still unknown. Based on our previous findings, we proposed a possible protective function of BACE1 in the pathogenesis of acute pancreatitis. To address this hypothesis, we induced acute pancreatitis in homozygote *Bace1* ^*-/-*^ and wild type mice by intraperitoneal injection of caerulein. Loss of Bace1 does not alter the severity of experimental caerulein-pancreatitis in mice. Thus, further studies are needed to clarify the physiological role of BACE1 in the exocrine pancreas.

## Materials and Methods

### Ethics statement

All animal experiments were performed according to the Guide for the Care and Use of Laboratory Animals published by the US National Institutes of Health (NIH Publication No. 85–23, revised 1996). The local ethics committee (Regierungspräsidium Leipzig) of the state of Saxony, Germany specifically approved this study (animal experiment registration number TVV 07/06 and TVV 03/14).

### Animals

*Bace1* ^*-/-*^ mice with a C57Bl/6 background (strain name: B6.129-*Bace1tm1Pcw*/J; 10–12 weeks) were supplied by Jackson Laboratory (Sacramento, USA). Age- and sex matched C57BL/6 mice served as wild type controls and were received from Medizinisch-Experimentelles Zentrum (MEZ), Universität Leipzig.

### Caerulein-induced pancreatitis

Mice were fasted for 12 h with free access to water. Acute pancreatitis was induced by 7 hourly intraperitoneal (i.p.) injections of caerulein (SIGMA, St. Louis, USA) at a dosage of 50 μg/kg body weight. Controls received 0.9% sterile saline. Mice were sacrificed at three different time points. T1 = four hours after the first injection (saline: *Bace1* ^*-/-*^ n = 5 [m = 5], C57BL/6 n = 5 [m = 5]; caerulein: *Bace1* ^*-/-*^ n = 5 [m = 5], C57BL/6 n = 5 [m = 5]). T2 = eight hours after the first injection (saline: *Bace1* ^*-/-*^ n = 5 [m = 2, f = 3], C57BL/6 n = 7 [m = 3, f = 4]; caerulein: *Bace1* ^*-/-*^ n = 6 [m = 3, f = 3], C57BL/6 n = 7 [m = 3, f = 4]). T3 = 24 hours after the first injection (saline: *Bace1* ^*-/-*^ n = 5 [m = 5], C57BL/6 n = 5 [m = 5]; caerulein: *Bace1* ^*-/-*^ n = 5 [m = 5], C57BL/6 n = 5 [m = 5]). During the phase of induction, access to food was denied. Mice were sacrificed according to the guidelines of the University of Leipzig Animal Care Committee for subsequent analysis. Serum samples were obtained at the beginning of the experiment and 4, 8 and 24 h after the first injection.

### Amylase and lipase measurement

For measurement of α-amylase and lipase, blood was collected by retroorbital bleeding before the first and 4, 8 and 24 hours after the first caerulein injection. Measurement of plasmatic amylase and lipase was performed in mice-adjusted instrument settings at the Institute of Laboratory Medicine, Clinical Chemistry and Molecular Diagnostics, of University Hospital Leipzig. Enzyme activities of amylase and lipase within plasma samples, collected 4, 8 and 24 hours after the first caerulein injection, were routinely measured on a Hitachi Modular Analytics (Roche Diagnostics, Mannheim, Germany).

### Hematoxylin & eosin staining

Pancreatic tissue was removed from euthanized mice, fixed in 4% paraformaldehyde (Roth, Karlsruhe, Germany) for 18 h and embedded in paraffin (Medite, Burgdorf, Germany). Samples were cut (5μm; Microm HM355S, Thermo Scientific, Walldorf, Germany), mounted and dried overnight at 40°C. Tissue sections were stained with hematoxylin (Medite, Burgdorf, Germany) and eosin (Medite, Burgdorf, Germany) using routine procedures.

### Immunohistochemistry

For immunohistochemical detection of myeloperoxidase, pancreatic tissue sections were de-paraffinized and incubated at 85°C in target retrieval solution pH 9 (Dako, Glostrup, DK) for 15 min. After blocking of endogenous peroxidase using 0.3% hydrogen peroxide (Roth, Karlsruhe, Germany) and incubation with 5% donkey serum (SIGMA, St. Louis, USA), a polyclonal rabbit anti-MPO antibody (Abcam, Cambridge, UK) was applied in a dilution of 1:200 in 2% donkey serum for 30 min. Detection was carried out using an anti-rabbit peroxidase (POD)-labeled antibody (Dianova, Hamburg, Germany). Liquid DAB^+^ substrate chromogen (Dako, Glostrup, DK) was applied for 7 min to visualize the immunostaining. Counterstaining was performed using hematoxylin. For immunohistochemical detection of Bace2 a polyclonal rabbit anti-Bace2 antibody (Abcam, Cambridge, UK) was used in a dilution of 1:50.

### Image recording and analysis

Bright field images were recorded on a BZ-8000 microscope (Keyence, Osaka, Japan) using a PlanApo objective (20x/0.75; Nikon, Melville, USA) at 200x and 400x magnification. BZ viewer and Analyzer 2.5.1 software were applied. The image resolution was 1,360 x 1,024 pixel (24 bit depth). The number of neutrophiles was determined manually using SigmaScan 5.0 software (Systat Software, San Jose, USA) by counting MPO^+^ cells in ten high power fields (200x) per mouse. Images were recorded at sides of most focal inflammation to reduce bias.

H&E sections of pancreatic tissues were assessed at 200x magnification over ten separate fields for severity of pancreatitis. Evaluation of edema, inflammatory infiltration and apoptosis was performed with a three point scoring system (edema—0: absent or rare, 1: in the interlobular space, 2: in the intralobular space, 3: the isolated-island shape of pancreatic acinus; inflammation—0: absent, 1: mild (infiltration in ducts), 2: moderate (infiltration in parenchyma < 50%), 3: severe (infiltration in parenchyma > 50%); parenchyma apoptosis—0: absent, 1: focal (<5%), 2: and/or sublobular (<20%), 3: and/or lobular (>20%)). Analysis was performed in a blinded manner.

### Reverse transcription quantitative real-time PCR (RT-qPCR)

Whole pancreatic tissue from *Bace1* ^*-/-*^ and C57BL/6 mice was frozen in liquid nitrogen and mortared. Total tissue mRNA was extracted using TRIzol Reagent (Invitrogen, Darmstadt, Germany) and RNeasy Mini Kit (Qiagen, Valencia, CA, USA) following the manufacturers instructions. A single step RT-qPCR was performed in a Light cycler 2.0 (Roche, Mannheim, Germany) with QuantiTect SYBR Green RT-PCR Kit and QuantiTect Primer Assay (both Qiagen). Primers for *Bace2* and *Gapdh* were from Qiagen. Sequences of the primers were not available.

### Statistics

Data are given as mean ± SD unless otherwise stated. Mann-Whitney U tests were applied for testing of differences between groups (Prism 5, GraphPad, San Diego, USA). Values of p < 0.05 were regarded as significant.

## Results

We previously reported that the protease Bace1 is expressed in the murine exocrine pancreas and detectable in pancreatic juice. Enteropeptidase was identified as one putative substrate for pancreatic Bace1 *in vitro* [17]. Based on these findings, we postulated a possible protective function of Bace1 against the development of acute pancreatitis by counteracting premature trypsinogen activation via enteropeptidase.

**Fig 1:**
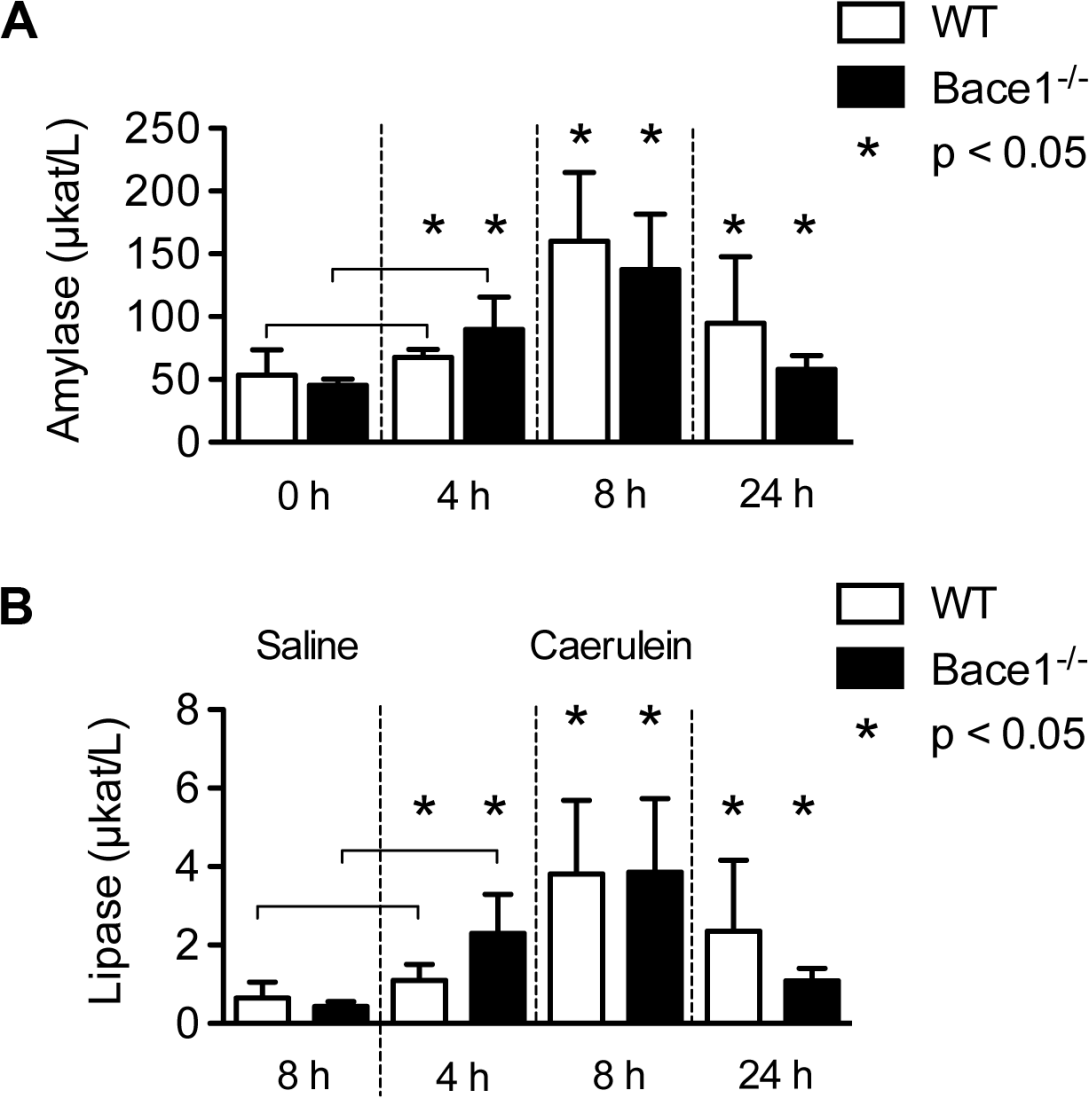
Plasmatic amylase and lipase measurement. **(A)** Plasmatic amylase activity was measured before induction of acute pancreatitis and after four, eight and 24 hours. Intraperitoneal application of caerulein led to elevated levels of amylase with the highest peak after eight hours. Asterisks denote significant differences (p < 0.05) compared to time point 0 h. **(B)** Lipase activity was measured four, eight and 24 hours after induction of acute pancreatitis. No increase of lipase activity could be detected in C57BL/6 and Bace1^-/-^ mice receiving sterile saline. Intraperitoneal application of caerulein led to significantly elevated levels of lipase. Each group consisted of at least five C57BL/6 and *Bace1* ^*-/-*^ mice. Asterisks denote significant differences (p < 0.05) compared to the saline groups. Measured activity is shown in μkat/L (mean ± SD). WT = wild-type (C57BL/6).

To determine the role of Bace1 in acute experimental pancreatitis, we analyzed the onset of inflammation in *Bace1* ^*-/-*^ mice in comparison to age- and sex-matched C57BL/6 mice. Acute pancreatitis was induced by intraperitoneal injection of caerulein. Controls received sterile saline. We performed tissue and blood analysis 4, 8 and 24 hours after the first caerulein injection. We assessed the onset of acute pancreatitis by measurement of plasmatic amylase and lipase. Amylase activity in the caerulein-group before the first injection was 64.8 ± 27.2 μkat/L in wild type and 48.4 ± 5.8 μkat/l in *Bace1* ^*-/-*^ mice. Already after 4 h, WT controls (67.0 ± 6.5 μkat/L) and *Bace1* ^*-/-*^ mice (89.8 ± 25.9 μkat/L) had an increased level of α-amylase. After 8 h, WT controls (160.0 ± 55.0 μkat/L) and *Bace1* ^*-/-*^ mice (137.5 ± 44.3 μkat/L) showed a significant increase of α-amylase. In addition, elevated plasmatic lipase activity in the caerulein-group (*Bace1* ^*-/-*^ mice (3.9 ± 1.9 μkat/L) and WT mice (3.8 ± 1.9 μkat/L)) was detected after 8 hours, indicating a successful induction of pancreatitis. 24 hours after the first caerulein injection serum levels of amylase and lipase were significantly decreased in both groups (Fig 1). As expected, no changes of amylase and lipase levels were detected in the saline-groups (data not shown). Measurement of plasmatic bilirubin, gamma-glutamyltransferase, alkaline phosphatase and glucose revealed matching results in both groups (data not shown).

**Fig 2:**
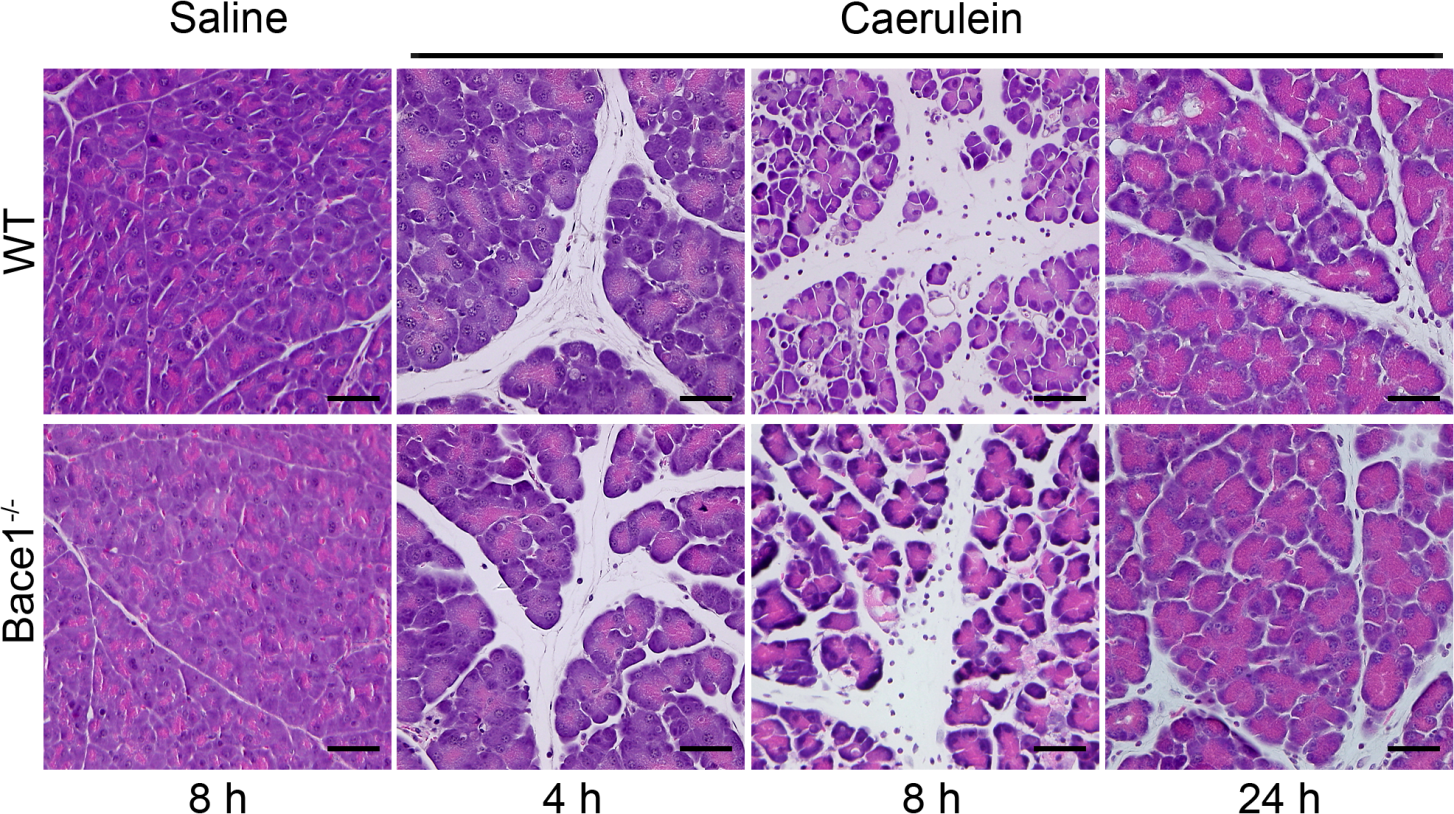
H&E histology images after pancreatitis induction. Acute pancreatitis in wild type and *Bace1* ^*-/-*^ mice was induced by caerulein. Pancreatic tissue was obtained four, eight and 24 hours after the first caerulein injection. Representative images of H&E stained pancreatic tissue sections at different time points are shown (magnification 200x). *Bace1* ^*-/-*^ mice show similar histomorphological features of acute pancreatitis like interlobular edema and inflammation compared to wild type mice. Scale bar = 50 μm. WT = wild type (C57BL/6).

For histological analysis pancreatic tissue sections were stained with hematoxylin and eosin. Representative histology images for wild type and *Bace1* ^*-/-*^ mice treated with saline or caerulein are shown in Fig 2. We observed no features of pancreatitis after the treatment with saline, whereas a moderate pancreatitis occurred within 8 hours after the first caerulein injection that diminished after 24 hours. Notably, we detected no significant difference between pancreatitis in *Bace1* ^*-/-*^ and wild type mice.

**Fig 3:**
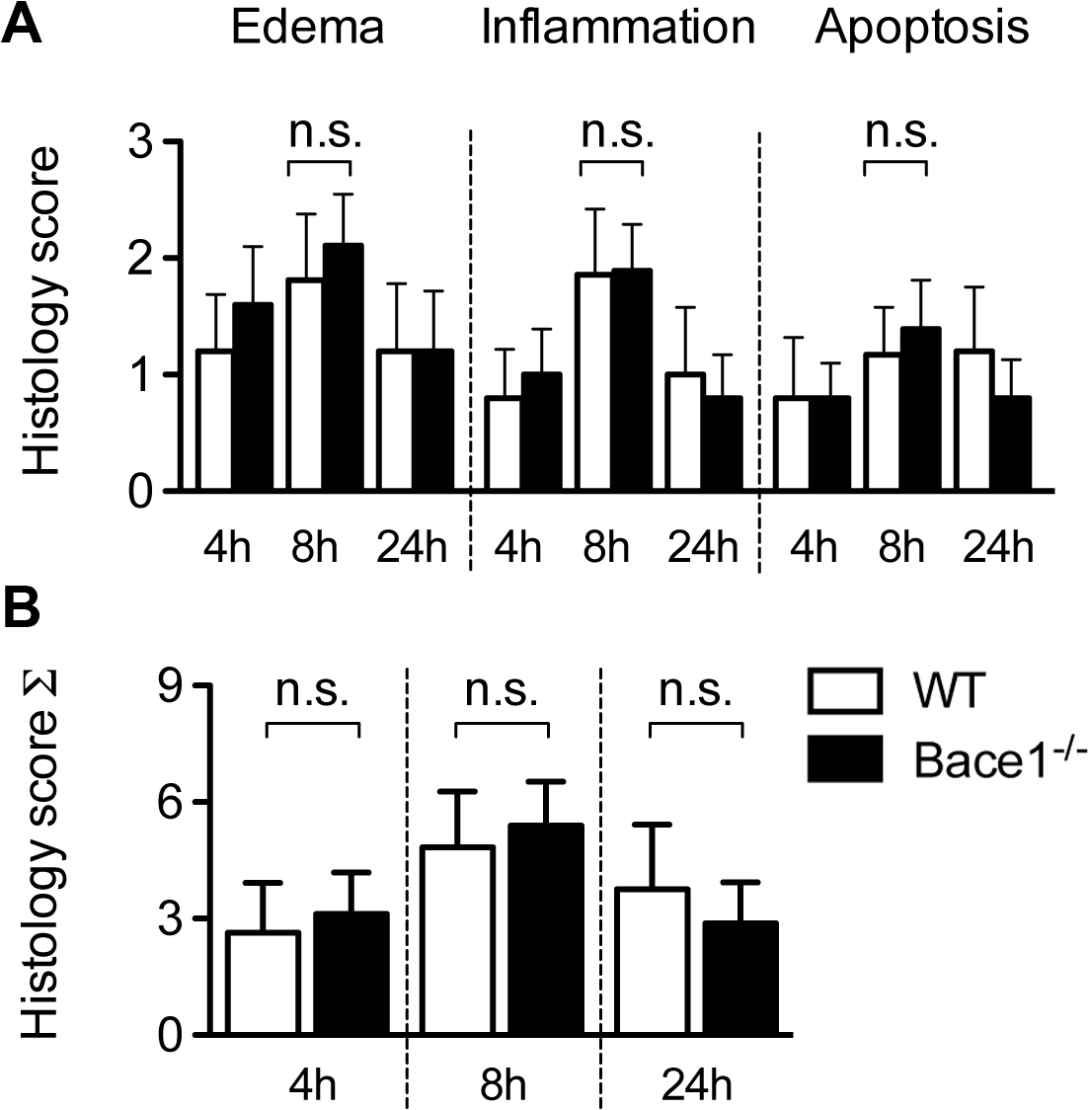
Histology score. **(A)** Pancreatic tissue sections of *Bace1* ^*-/-*^ and wild-type mice were scored for severity of pancreatitis after four, eight an 24 hours following the first cearulein injection. Histology scores for pancreatic edema, inflammation and apoptosis are shown and were generated by using a three point scoring system (0 = absent, 1 = mild, 2 = moderate, 3 = severe). *Bace1* ^*-/-*^ mice as well as wild-type mice show moderate edema, moderate inflammation and focal apoptosis within the parenchyma during the course of disease. These features are most prominent eight hours after the first injection and already regressing after 24 hours. **(B)** Total histology score is shown as mean ± SD. Each group consisted of at least five C57BL/6 and *Bace1* ^*-/-*^ mice. n.s. = not significant. WT = wild type (C57BL/6).

H&E stained pancreatic tissue sections were scored for severity of pancreatitis by evaluating the extent of edema, inflammation and apoptosis during the course of pancreatitis at the different time points (Fig 3). To this end, we used a three point scoring system (0 = absent, 1 = mild, 2 = moderate, 3 = severe) as previously described [18]. Already after 4 hours we detected a mild edema within the pancreas of *Bace1* ^*-/-*^ (n = 5) and WT (n = 5) mice, whereas inflammation and apoptosis was almost absent. After 8 hours, both groups showed edema in the interlobular as well as intralobular space (WT 1.8 ± 0.5 (n = 7); *Bace1* ^*-/-*^ 2.1 ± 0.4 (n = 6)). In addition, we observed a moderate inflammation of the pancreas in *Bace1* ^*-/-*^ as well as in the wild type controls. Inflammatory cells were mostly located in the interlobular space and to a minor amount in the parenchyma. The extent of parenchyma infiltration was on average less then 50%. It has to be noted that no significant differences of inflammation could be detected in the caerulein-group (WT 1.9 ± 0.6; *Bace1* ^*-/-*^ 1.9 ± 0.4). Pancreatic sections of wild type and *Bace1* ^*-/-*^ mice showed apoptotic cells in less then 5 to 20% of the parenchyma (WT 1.2 ± 0.4; *Bace1* ^*-/-*^ 1.4 ± 0.4) and furthermore, no necrosis could be detected. Within 24 hours, we observed a decrease of edema (WT 1.2 ± 0.6 (n = 5); *Bace1* ^*-/-*^ 1.2 ± 0.5 (n = 5)) and inflammation (WT 1.0 ± 0.6; *Bace1* ^*-/-*^ 0.8 ± 0.4).

We calculated a total score of the single experiments in order to minimize alterations in tissue samples. Caerulein-treated mice developed a moderate pancreatitis with a peak after 8 hours, but we observed no significant difference between the *Bace1* ^*-/-*^ mice and the wild type controls during the course of disease (Fig 3). *Bace1* ^*-/-*^ mice and wild type controls treated with sterile saline showed no histological signs of pancreatitis (data not shown). The analysis of the H&E stained pancreatic tissue sections showed that loss of Bace1 led to no significant changes in the development of acute experimental pancreatitis.

**Fig 4:**
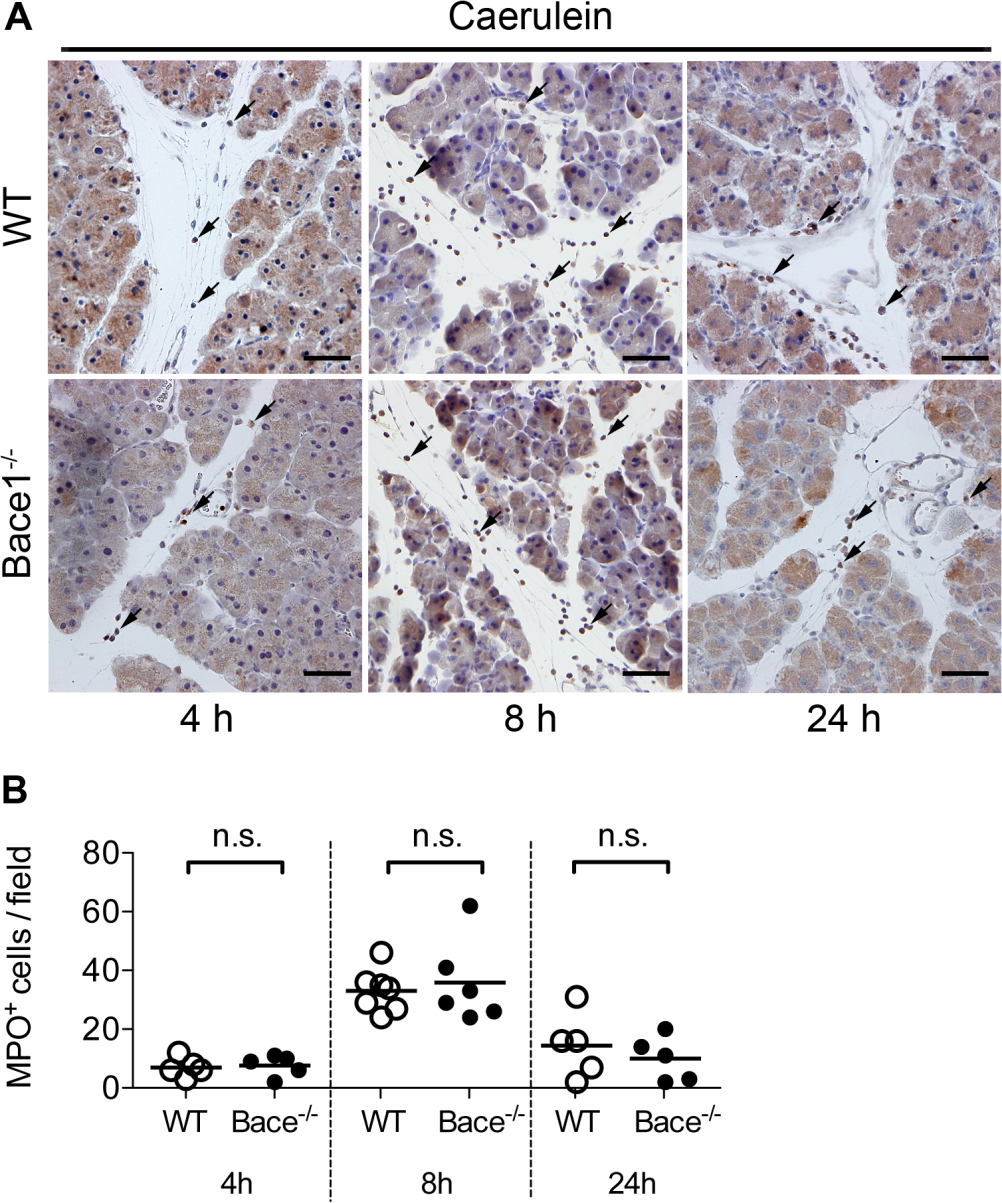
Immunohistochemical detection of myeloperoxidase. **(A)** Detection of myeloperoxidase (MPO) by immunohistochemistry in pancreatic tissue sections four, eight and 24 hours after induction of acute pancreatitis in wild-type C57BL/6 and *Bace1* ^*-/-*^ mice. Representative images are shown in 200x magnification. A polyclonal rabbit antibody (anti-MPO, Abcam) was used in an optimized dilution of 1:200 to keep unspecific signals within the parenchyma to a minimum. Staining was visualized using DAB. Arrows indicate MPO positive inflammatory cells. Scale bar represents 50 μm in all images. **(B)** MPO positive cell counts per field (200x) are shown for wild type and *Bace1* ^*-/-*^ mice during the course of disease. Symbols represent individual mice. Line represents mean. n.s. = not significant.

To confirm these observations, we quantified the inflammation at different time points by immunohistochemical detection of myeloperoxidase (MPO), a marker for neutrophile granulocytes. We detected MPO positive cells mainly within the inter- and intralobular space (Fig 4). In accordance to the previous findings, *Bace1* ^*-/-*^ and wild type mice showed a similar number of MPO positive cells per high power field after 8 hours (WT 33 ± 7 MPO+ cells/field; *Bace1* ^*-/-*^ 36 ± 14 MPO+ cells/field). After 24 hours, we found a significant reduction of MPO+ cells (WT 14 ± 11 MPO+ cells/field; *Bace1* ^*-/-*^ 10 ± 7 MPO+ cells/field) but no differences between *Bace1* ^*-/-*^ and WT mice (Fig 4B). Thus, targeted disruption of *Bace1* has no influence on the extent of inflammation in acute experimental pancreatitis compared to wild type controls.

**Fig 5:**
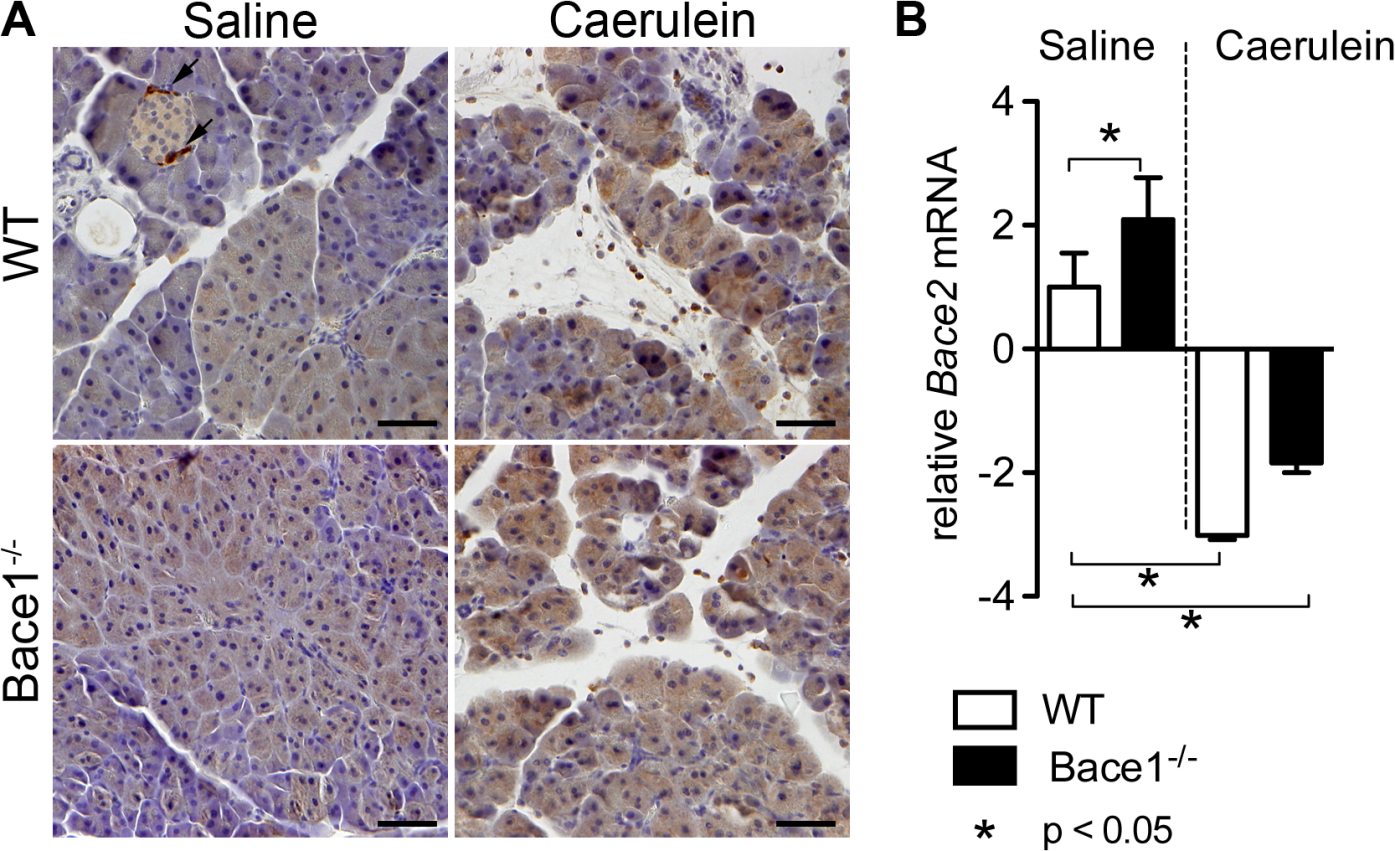
Immunohistochemical detection of Bace2 and RT-qPCR of *Bace2* mRNA in pancreatic tissue. **(A)** Detection of Bace2 by immunohistochemistry in pancreatic tissue sections eight hours after the first cearulein injection in wild-type C57BL/6 and *Bace1* ^*-/-*^ mice. Control groups received sterile saline. Bace2 is expressed within the acinar cells of wild-type C57BL/6 and *Bace1* ^*-/-*^ mice. There is a slightly higher signal for Bace2 during acute pancreatitis but no difference between WT and *Bace1* ^*-/-*^ mice. Bace2 enriched alpha cells of the endocrine pancreas served as internal positive controls. Representative images are shown in 200x magnification. A polyclonal rabbit antibody (anti-Bace2, Abcam) was used in a dilution of 1:50. Staining was visualized using DAB. Arrows indicate positive alpha cells within the islets. Scale bar represents 50 μm in all images. **(B)** RT-qPCR of *Bace2* mRNA in whole pancreatic tissue of wild-type C57BL/6 and *Bace1* ^*-/-*^ mice eight hours after the first injection of caerulein or sterile saline (n = 4 in each group). Pancreatic *Bace2* mRNA levels were 2.1 fold higher in *Bace1* ^*-/-*^ mice as compared to wild-type C57BL/6 mice of the saline group (p<0.05). During caerulein pancreatitis Bace2 mRNA expression is significantly reduced in both C57BL/6 and *Bace1* ^*-/-*^ mice as compared to wild-type C57BL/6 mice of the saline group (p<0.05). Results are expressed as fold induction to wild-type C57BL/6 mice of the saline group using *Gapdh* as a housekeeping gene and are the means ± SD.

We speculated that Bace2, another ß-secretase homolog with a high similarity to Bace1 and high expression within the pancreas might be able to compensate the loss of Bace1. Therefore, we examined for differences in Bace2 expression in Bace1^-/-^ mice compared to wild type controls. Immunohistochemical analysis showed that Bace2 is expressed overall within the acinar cells of wild-type C57BL/6 and *Bace1* ^*-/-*^ mice. We could find a slightly higher signal for Bace2 during acute pancreatitis but no significant difference between WT and *Bace1* ^*-/-*^ mice (Fig 5A).

Expression of *Bace2* mRNA in whole pancreatic tissue of wild-type C57BL/6 and *Bace1* ^*-/-*^ mice was measured by RT-qPCR. Pancreatic *Bace2* mRNA levels were 2.1 fold higher in *Bace1* ^*-/-*^ mice as compared to wild-type C57BL/6 mice of the saline group (p<0.05). During caerulein pancreatitis Bace2 mRNA expression was significantly reduced in both C57BL/6 and *Bace1* ^*-/-*^ mice as compared to wild-type C57BL/6 mice of the saline group (Fig 5B).

## Discussion

The aspartic protease BACE1 is primarily expressed within the central nervous system by neurons and has been connected to the pathogenesis of Alzheimer’s disease [12]. In addition, BACE1 finsertms to play a role in glucose and lipid homoeostasis and may be a link between Alzheimer’s disease and diabetes [19, 20]. Besides its well-characterized expression pattern in the brain, BACE1 mRNA was also detected in non-neuronal tissue, especially within the pancreas [21, 22]. Although, pancreatic ß-cells are the main source of BACE1 mRNA [23], we previously reported that Bace1 is expressed in exocrine pancreatic acinar cells as well [17]. Immunohistochemical analysis revealed that Bace1 is mainly located at the apical pole of acinar cells in mice, emphasizing a role for Bace1 either within the process of enzyme secretion or secretion of Bace1 into the pancreatic juice itself. The later was partly proofed by detection of Bace1 activity within the pancreatic juice. Additionally, we found enteropeptidase to be a putative substrate for recombinant BACE1 *in vitro* [17]. Based on these observations, we proposed an involvement of BACE1 in a defense mechanism during development of acute pancreatitis. To address, if a loss of Bace1 leads to an increased severity of acute pancreatitis, we induced pancreatitis in *Bace1* ^*-/-*^ mice by intraperitoneal caerulein-injections. However, targeted disruption of *Bace1* in mice had no influence on the development and severity of acute experimental pancreatitis on the basis of histological, immunohistochemical and serological findings.

Both, *Bace* ^*-/-*^ and wild type mice showed moderate histomorphological signs of edematous pancreatitis with a peak at 8 hours after the first caerulein injection. The increase of plasmatic amylase and lipase levels indicated a pancreatitis induction and the determined histology scores are consistent with those found in the literature [24]. We quantified the number of myeloperoxidase positive cells within the pancreas to identify small differences regarding the extent of inflammation. In accordance with the data collected from the H&E staining, we found no differences between *Bace1* ^*-/-*^ and wild type mice.

It is believed that acute pancreatitis occurs on the basis of premature intra-acinar trypsinogen activation, followed by a trypsin driven cascade of zymogen activation leading to autodigestion of the gland [25]. Although a number of putative substrates for Bace1 other than alpha-amyloid protein have been identified *in vitro* using quantitative proteomics [26], still nothing is known about physiological substrates within the exocrine pancreas. Nevertheless, we speculated that Bace1 could protect the pancreas against premature trypsinogen activation by cleavage of activated zymogens or enteropeptidase derived from duodenopancreatic reflux. Occasionally occurring duodenopancreatic reflux had been proposed as a cause of acute pancreatitis [27]. The widely accepted model of caerulein induced pancreatitis is an experimental pancreatitis model based on acinar hyperstimulation and might not quite resemble the pathophysiology regarding duodenopancreatic reflux. In previous reports, this model was described to lead to hyperstimulation of acinar cells which causes a blockage of enzyme secretion. Furthermore, co-localization of zymogens and lysosomal enzymes, activation of trypsinogen and acinar cell injury were all alterations seen after caerulein stimulation [28]. Additional intravenous application of enteropeptidase to i.p. injection of caerulein caused a severe necrotizing pancreatitis [29]. However, the latter model seems to be far away from physiological processes. Other pancreatitis models like bile–pancreatic duct obstructions or retrograde taurolithocholic acid 3-sulfate injections have been shown to be very invasive and to produce varying results [30]. Thus, the caerulein-pancreatitis used in this study seems the most suitable model for general observation. Although, we could detect no significant differences between *Bace1* ^*-/-*^ and wild type mice, our data show a tendency for *Bace1* ^*-/-*^ mice to develop slightly increased edema and inflammation especially in the early stage (4 h) and after 8 hours. Since caerulein-induction over 8 hours causes only a mild edematous pancreatitis small changes in the severity are more difficult to detect, which could possibly be compensated by a more severe model or larger animal numbers.

With our findings we can assume, that Bace1 might not be directly involved in the process of acute experimental caerulein-pancreatitis in mice. We speculated that Bace2, another ß-secretase homolog with a high similarity to Bace1, might be able to compensate the loss of Bace1. Interestingly, Bace2 mRNA is expressed in many embryonic and adult tissues, but the highest level of expression was found in adult pancreas [31]. The increased neonatal mortality seen in *Bace1/Bace2* double knockout mice compared with single *Bace1* knockout could be an indication that some overlap between Bace1 and Bace2 functions exists [32]. Immunohistochemical analyses showed no significant up-regulation of Bace2 in the pancreas of *Bace1* ^*-/-*^ mice. On the mRNA level we could detect a 2fold expression of *Bace2* mRNA in the pancreas of *Bace1* ^*-/-*^ mice compared to controls. Since there are no clear data on the functional role of either Bace1 or Bace2 within the exocrine pancreas the biological relevance of the found overexpression of Bace2 mRNA remains highly speculative. Hence, further investigations focusing on BACE and its relevance for the pancreas and pancreatic diseases need to be carried out.
